# Psychological and Pharmacological Interventions to Reduce Alcohol Use Disorder (AUD) in the inpatient units. A General Review.

**DOI:** 10.1192/j.eurpsy.2024.567

**Published:** 2024-08-27

**Authors:** E. Owusu, R. Shalaby, N. Nnamdi, L. A. Mobolaji, V. I. Agyapong

**Affiliations:** ^1^Psychiatry, University of Alberta, Edmonton; ^2^Psychiatry, Dalhousie University, Halifax, Canada

## Abstract

**Introduction:**

According to the World Health Organization, around 2 billion people worldwide are estimated to drink. Alcohol intake results in 25% of the 3.8% of worldwide fatalities and 4.6% of global disability-adjusted life years that may be attributed to alcohol

**Objectives:**

This review seeks to synthesize data on psychological and pharmacological treatments for Alcohol Use Disorder (AUD) available in the inpatient setting.

**Methods:**

A comprehensive and narrative review of studies and research on psychological and pharmacological interventions for patients with alcohol use disorders in inpatient treatment units was performed. Data was extracted from electronic bibliographic databases, including Medline, EMBASE, PsycINFO, Global Health, HealthSTAR, and Cumulative Index for Nursing and Allied Health Literature (CINAHL) via EBSCOhost. This review included both qualitative and quantitative studies

**Results:**

Overall, after an initial title, abstract screening, and subsequent full-text screening, seven out of 1245 extracted studies met the eligibility criteria and were included in the review. This review suggests that a combination of pharmacological interventions such as naltrexone, nalmefene, acamprosate and brief psychological interventions were effective in treating AUD.

**Conclusions:**

This review suggests that pharmacological and psychological approaches, when used together, are efficacious in treating AUD. There is a need to adopt both pharmacological and psychological interventions in the treatment of AUD.

**Disclosure of Interest:**

None Declared

